# Acute Neurological Presentation in Children With SARS-CoV-2 Infection

**DOI:** 10.3389/fped.2022.909849

**Published:** 2022-07-11

**Authors:** Antonella Riva, Gianluca Piccolo, Federica Balletti, Maria Binelli, Noemi Brolatti, Alberto Verrotti, Elisabetta Amadori, Alberto Spalice, Thea Giacomini, Maria Margherita Mancardi, Paola Iannetti, Maria Stella Vari, Emanuela Piccotti, Pasquale Striano, Giacomo Brisca

**Affiliations:** ^1^Department of Neurosciences, Rehabilitation, Ophthalmology, Genetics, Maternal and Child Health, University of Genoa, Genoa, Italy; ^2^Pediatric Neurology and Muscular Diseases Unit, IRCCS Istituto Giannina Gaslini, Genoa, Italy; ^3^Department of Pediatrics, University of Perugia, Perugia, Italy; ^4^Department of Pediatrics, Child Neurology Division, Sapienza University Rome, Rome, Italy; ^5^Child Neuropsychiatry Unit, IRCCS Istituto Giannina Gaslini, Genoa, Italy; ^6^Department of Paediatrics, University of Rome, Rome, Italy; ^7^Pediatric Emergency Department, IRCCS Istituto Giannina Gaslini, Genoa, Italy; ^8^Subintensive Care Unit, IRCCS Istituto Giannina Gaslini, Genoa, Italy

**Keywords:** acute, COVID-19, children, neurological symptoms, SARS-CoV-2

## Abstract

**Background:**

In the pediatric population, the knowledge of the acute presentation of SARS-CoV-2 infection is mainly limited to small series and case reports, particularly when dealing with neurological symptoms. We describe a large cohort of children with acute SARS-CoV-2 infection, focusing on the neurological manifestations and investigating correlations between disease severity and population demographics.

**Methods:**

Patients aged 0–18 years with a positive molecular swab were recruited between April 2020 and March 2021 from a tertiary Italian pediatric centre. Clinical data, imaging, and laboratory test results were retrieved from our local dataset and statistically analyzed.

**Results:**

A total of 237 patients with a median age of 3.2 years were eligible; thirty-two (13.5%) presented *with* neurological symptoms, including headache (65.6%), altered awareness (18.8%), ageusia/anosmia (12.5%), seizures (6.3%), and vertigo (6.3%), combined in 7 (21.9%) cases. Respiratory (59.5%) and gastrointestinal (25.3%) symptoms were the most common among the 205 (86.5%) patients *without* neurological involvement. Neurological symptoms did not significantly influence the severity of the triage access codes. Moreover, pre-existing medical conditions were not higher in the group *with* neurological manifestations. Overall, fifty-nine patients (25%, 14/59 *with* neurological symptoms) required treatment, being antibiotics, systemic steroids, and heparin those most prescribed.

**Conclusion:**

Our study supports the overall benign course of the SARS-CoV-2 infection in children. Neurological manifestations, except for headache, remain a rare presenting symptom, and disease severity seems unrelated to pre-existing medical conditions.

## Introduction

Severe acute respiratory syndrome coronavirus 2 (SARS-CoV-2) is the human coronavirus responsible for the Coronavirus disease 2019 (COVID-19) pandemic, which spread worldwide starting from late 2019 to early 2020 (1). Likewise, its “cousins” [i.e., SARS-CoV and the Middle East respiratory syndrome (MERS-CoV)], SARS-CoV-2 can replicate in the epithelial cells and pneumocytes of the lower respiratory tracts, causing either pneumonia or acute respiratory distress syndrome (2–4). However, the clinical spectrum of COVID-19 is largely heterogeneous and disease severity and progression are mainly influenced by host factors, including age, sex, and pre-existing chronic conditions (e.g., hypertension, type 2 diabetes mellitus, and obesity) (5–10). Particularly, current evidence suggests that age itself is the most significant risk factor for severe COVID-19 and its adverse health outcomes (11).

To date, few studies have specifically investigated the acute neurological presentation of COVID-19 in the pediatric population. The rate of asymptomatic children ranges from 4.4 to 23% of all the cases, and may be undermined as many asymptomatic children escape screening (12–16). The most frequent non-neurological manifestations are fever, cough, respiratory distress, rhinorrhea, sneezing or nasal congestion, pharyngitis, vomiting or nausea, abdominal pain, diarrhea, and fatigue (17). Factors associated with intensive care unit (ICU) admission are mainly represented by neonatal age, male gender, lower respiratory tract disease, and pre-existing medical conditions (e.g., chronic pulmonary disease, congenital heart disease, malignancies, and neurological disorders) (14, 15). Mortality rate in pediatric cohorts is low (up to 0.7%) (12–14, 18, 19).

Neurological manifestations of COVID-19 in children are mainly limited to headache, asthenia, and ageusia/anosmia, the latter being particularly difficult to assess in this population and, thus, underreported (16, 20–23). However, more severe neurological complications, including encephalitis, seizures, and cerebrovascular infarct, are described in small series or single case reports (23–32).

Additional research is needed to fully assess the neurological implications of the SARS-CoV-2 infection in children. We report the clinical presentation of a large cohort of children whit acute SARS-CoV-2 infection, describing the neurological features, as well as investigating correlations between disease severity and population demographics.

## Methods

### Patients

Patients aged 0–18 years who tested positive for SARS-CoV-2 with a molecular swab at the Emergency Department (ED) or on admission to a ward, were recruited from a tertiary Italian pediatric centre between April 2020 and March 2021. Clinical data including previous medical history, imaging, and laboratory test results were retrospectively collected through our local dataset. Patients’ parents/caregivers gave written informed consent. The study was reviewed and approved by Comitato Unico Regionale Regione Liguria, Genova, Italy.

### Statistical Analysis

Patients were divided into two groups; those *with* and those *without* neurological symptoms. Categorical variables were compared using the Chi-squared test (X^2^) if expected frequencies > 5, otherwise using the Fisher’s exact test. The thresholds of *p*-value were set at 0.05 (statistical significance) and 0.01 (high statistical significance). Quantitative variables were reported in terms of mean values and standard deviations (SD) in the case of normally distributed data (determined using the Shapiro–Wilk test) or in terms of median values with 1st and 3rd quartiles (1st–3rd q) in case of skewed distributions. Mann–Whitney *U* test was used to compare two quantitative variables in case of skewed distributions. Each neurological manifestation was further stratified by age in two subgroups: pre-scholar (<6 years) and scholar (>6 years).

## Results

### Clinical Features

A total of 237 patients (113 females) were recruited (Table 1). The median age was 3.2 years (0.8–10.7 years, 1st–3rd q). Two hundred twenty-two (93.7%) patients were tested at the ED, of which 182 (76.8%) were admitted with symptoms suggestive of COVID-19 (i.e., fever, cough, pharyngodynia, rhinitis, headache, vomiting, and diarrhea). Forty-three (18%) individuals were asymptomatic, 15 (34.9%) of them being positive at a scheduled admission to the ward.

**TABLE 1:** Comparison between patients *with* and *without* neurological symptoms.

	Patients *with* neurological symptoms	Patients *without* neurological symptoms	*p*-value
Total, *n* (%)	32 (13.5)	205 (86.5)	
Female, *n* (%)	18 (56.25)	95 (46.34)	0.3436
Ethnicity: Caucasian, *n* (%)	26 (81.25)	143 (69.76)	0.2123
Median age, y (1st–3rd q)	10.9 (5.8–13.3)	2.5 (0.7–9)	**0.0002**
Respiratory symptoms, *n* (%)	21 (65.63)	122 (59.51)	0.5646
GI symptoms, *n* (%)	17 (53.13)	52 (25.37)	**0.0018**
Cardiologic symptoms, *n* (%)	1 (3.13)	6 (2.93)	1
Hospitalized, *n* (%)	14 (43.75)	88 (42.93)	1
Mean admission duration, d	6	4	–
Pre-existing conditions, *n* (%)	7 (21.8)	37 (18.05)	0.62

*d, days; GI, gastrointestinal; n, number; y, years. Respiratory symptoms include: cough, pharyngodynia, pharyngitis, rhinitis, respiratory distress, apnoea, and chest pain. GI symptoms include: nausea, vomiting, abdominal pain, and diarrhea. Statistically significant p-value is in bold.*

**FIGURE 1:**
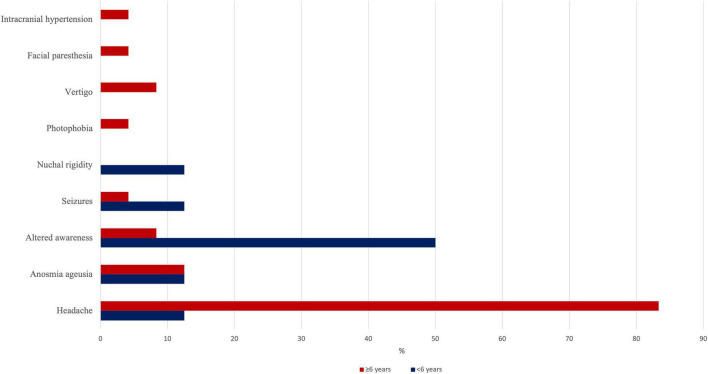
Prevalence of each neurological manifestation in our series, stratified by age subgroups (<6 or >6 years).

Thirty-two (13.5%) patients with a median age of 10.9 years (5.8–13.3 years) presented *with* neurological symptoms including headache (65.6%), altered awareness (18.8%), ageusia/anosmia (12.5%), seizures (6.3%), and vertigo (6.3%). Photophobia, facial paresthesia, endocranial hypertension, and meningitis were found in each patient (Figure 1). Seven (21.9%) patients showed two or more associated neurological features. In this group of patients, cough, pharyngitis, rhinitis, and diarrhea were the most frequently associated non-neurological manifestations. Fourteen (43.8%) patients within this group required hospitalization with a median stay of 5 days (mean, 6 days). One patient (7.1%) only required, first, non-invasive and, then, invasive ventilation support due to bilateral pneumonia and respiratory failure. Seven (21.9%) children had pre-existing medical conditions (i.e., trilinear cytopenia, X-fragile syndrome, hyperthyroidism, ischemic stroke, jejunum atresia, asthma, and schizophrenia).

Two hundred and five (86.5%) patients with a median age of 2.5 years (0.7–9 years) did not report neurological symptoms. Eighty-eight (42.9%) of them required hospitalization with a median stay of 5.5 days (mean, 4 days); moreover, 3 (3.4%) patients required non-invasive ventilation support due to low O_2_ saturation parameters. In this group, respiratory (59.5%) and/or gastrointestinal (GI; 25.3%) involvements were the most common. Thirty-seven (18.1%) children had pre-existing medical conditions, of which 9 (18%) involved the respiratory tract (i.e., asthma, allergy, and cystic fibrosis).

Of the total cohort, fifty-nine patients (25%, 14/59 *with* neurological symptoms) required treatment. The most prescribed drugs (32/59, 54%) were antibiotics (e.g., amoxicillin-clavulanic acid), systemic steroids, and heparin. Intravenous immunoglobulins, pulmonary surfactant, and inotropic drugs were administered to a patient with symptoms suggestive of meningitis at the ED and who, then, underwent invasive ventilation.

In our cohort, 5 (2.1%) children had a previous history of epilepsy, namely symptomatic epilepsies (1 arachnoid cyst and 1 astrocytoma), genetic generalized epilepsy, developmental epileptic encephalopathy, and epilepsy associated with X-fragile syndrome. In all cases, seizures were well-controlled with a mean of 1.6 (range: 1–3) anti-seizure medications (ASMs). ASMs included valproate (3 patients), clobazam (2 patients), levetiracetam (2 patients), and ethosuximide (1 patient). The only patient admitted with seizure re-exacerbation was an 11-year-old girl affected by X-fragile syndrome under levetiracetam monotherapy; she experienced a focal-onset febrile seizure with spontaneous resolution, no additional investigations were needed, and she was discharged without changes in her treatment regimen.

**FIGURE 2:**
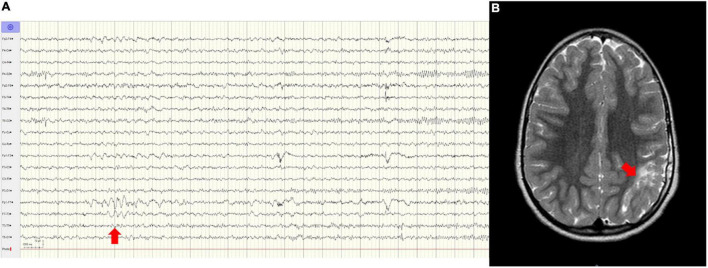
**(A)** EEG performed at the Emergency Department (ED) showing left fronto-temporal epileptiform abnormalities (arrow). **(B)** T2-weighted axial brain MR, showing the consequences of an old ischemic lesion in the left parietal lobe (arrow).

New-onset seizures occurred in a 5-year-old female with a history of ischemic stroke and jejunal atresia, admitted to the ED for a focal-onset motor seizure. Midazolam was administered with seizure remission. Brain magnetic resonance imaging (MRI) confirmed the previous ischemic lesion plus a post-ictal left fronto-insular perfusion alteration, and the electroencephalogram (EEG) showed left frontotemporal epileptiform abnormalities (Figure 2). The girl was discharged after 1 day in good clinical conditions without therapy.

### Imaging and Cardiological Findings

Imaging data were available in 61 (25.7%) patients; twenty-eight (45.9%) performed a chest X-ray in 16 (57.2%) cases showing interstitial pneumonia. Chest CT was performed in 4 patients, resulting in a case of microembolism and two of interstitial pneumonia. Chest MR performed on one patient showed hypoperfusion of the lower lung segments and a pleural effusion flap.

A brain CT scan was performed on the girl with epilepsy and a history of previous ischemic stroke, showing an unchanged ischemic area with left fronto-insular altered perfusion. This last finding was further confirmed with a brain MRI, and an EEG showing left fronto-temporal epileptiform abnormalities, compatible with the clinical presentation of a right-sided motor seizure. Additionally, other two patients performed a brain MRI: in one patient it was normal, whereas in the other case, it revealed *pseudotumor cerebri* in a child with papilledema at the ophthalmological examination.

Cardiac investigations (namely ECG, cardiac or epiaortic vessels ultrasounds) were performed in 13 (5.5%) patients, 8 *without* and 5 *with* neurological symptoms. In the group of patients *without* neurological symptoms, the main abnormal findings were altered cardiac rhythm at the ECG (i.e., tachycardia, and lower atrial rhythm alternating with sinus rhythm), mild mitral insufficiency, and “benign” pericardial effusion at the ultrasound. Conversely, among the 5 patients *with* neurological symptoms, there was a single case of thrombotic atrial formation.

### Laboratory Tests Results

Eighty-two (34.6%) patients underwent laboratory test assessment: blood cell count, inflammatory biomarkers including erythrocyte sedimentation rate (ESR), procalcitonin (PCT), ferritin, c-reactive protein (CRP), fibrinogen, D-Dimer, liver and kidney function, coagulation profile, and pro-BNP were evaluated when deemed clinically appropriate (Table 2). Median values of ferritin, CRP, fibrinogen, and D-dimer were similar between the two groups of patients (Table 3).

**TABLE 2:** Main laboratory test results in patients tested positive for severe acute respiratory syndrome coronavirus 2 (SARS-CoV-2).

Parameters (normal values)	Tested patients *n* (%)	Values out of normal age range *n* (%)
PCT (<0.50 ng/mL)	35 (14.7)	8 (22.9)
ESR (1–10 h/mm)	19 (8.0)	14 (73.7)
CRP (<0.46 mg/dL)	82 (34.5)	38 (46.3)
Fibrinogen (180–350 mg/dL)	41 (17.2)	21 (51.2)
aPTT (23.2–33.2 s)	39 (16.4)	2 (5.1)
PT% (63–129)	39 (16.4)	2 (5.1)
PT-INR (0.74–1.25)	34 (14.3)	0 (0.0)
D-dimer (<0.55 ug/mL)	26 (10.9)	19 (73.1)
Pro-BNP (0–125 pg/mL)	9 (3.8)	5 (55.6)
Troponins (0–0.16 ng/mL)	13 (5.5)	0 (0.0)
Glycemia (45–100 mg/dL)	73 (30.7)	23 (31.5)
AST (<40 U/L)	77 (32.4)	28 (36.4)
ALT (<40 U/L)	76 (31.9)	11 (14.5)
CK (0–150 U/L)	41 (17.2)	5 (12.2)
LDH (84–480 U/L)	49 (20.6)	28 (57.1)
Ferritin (20–200 ng/mL)	13 (5.5)	4 (30.8)
Haematocrit (36–44%)	82 (34.5)	5 (6.1)
Haemoglobin (11–13 g/dL)	85 (35.7)	34 (40.0)
Leucocytes (5,800–15,300/mm3)	86 (36.1)	2 (2.3)
Lymphocytes (31.9–73.1%)	84 (35.3)	5 (6.0)
Neutrophils (14.8–54.2%)	85 (35.7)	27 (31.8)
Platelets (150–400/mm3)	84 (35.3)	14 (16.7)

*n, number; CK, Creatin Kinase; CRP, C-Reactive Protein; ESR, Erythrocyte Sedimentation Rate; PCT, ProCalciTonin.*

**TABLE 3:** Comparison of inflammatory markers between patients *with* and *without* neurological symptoms.

	*With* neurological symptoms (median)	*Without* neurological symptoms (median)
Ferritin, ng/mL	115 (*n* = 3)	133.0 (*n* = 10)
PCT, ng/mL	0.25 (*n* = 7)	0.25 (*n* = 28)
CRP, mg/dL	0.23 (*n* = 12)	0.23 (*n* = 70)
Fibrinogen, mg/dL	327 (*n* = 5)	358 (*n* = 36)
D-dimer, mg/L	1.07 (*n* = 3)	1.21 (*n* = 23)

*n, number; CRP, C-Reactive Protein; PCT, ProCalciTonin.*

### Disease Severity-Population Demographics Correlations

**FIGURE 3:**
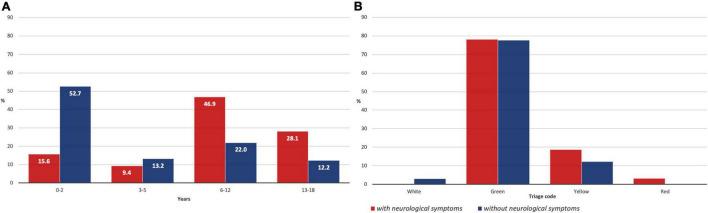
**(A)** Patients *with* and *without* neurological symptoms stratified by age range; **(B)** Patients *with* and *without* neurological symptoms stratified for priority triage code at the Emergency Department (ED).

Stratification by age range revealed a higher prevalence (46.9%) of neurological symptoms in patients aged between 6 and 12 years, followed by those aged 13–18 years (28.1%). A lower prevalence of neurological symptoms was found in patients younger than 6 years, whose symptoms mainly involve the respiratory or GI tract. Accordingly, the median age in the group *without* neurological symptoms was 2.5 years, compared to 10.9 years in patients *with* neurological symptoms (Supplementary Figure 1). The prevalence of each neurological manifestation stratified by age range is shown in Table 4 and Figure 3A.

**TABLE 4:** Patients *with* neurological symptoms stratified by age range.

AGE	Total pts N^°^ (%)	Headache	Anosmia/ageusia	Altered awareness	Seizures	Nuchal rigidity	Photophobia	Vertigo	Facial paraesthesia	Endocranial hypertension
**0–2 y**	5 (15.6%)	0	0	4 (80.0%)	0	1 (20.0%)	0	0	0	0
**3–5 y**	3 (9.4%)	1 (33.3%)	1 (33.3%)	0	1 (33.3%)	0	0	0	0	0
**6–12 y**	15 (46.9%)	12 (80.0%)	2 (13.3%)	2 (13.3%)	1 (6.7%)	0	1 (6.7%)	1 (6.7%)	0	1 (6.7%)
**13–18 y**	9 (28.1%)	8 (88.9%)	1 (11.1%)	0	0	0	0	1 (11.1%)	1 (11.1%)	0

The triage code given on admission at the ED according to the Italian former color code system (red – emergency, yellow – urgency, green – non-urgent, and white – minor issues) was green in 78.1%, yellow in 18.8%, and red in 3.1% of the children *with* neurological symptoms, while patients *without* neurological symptoms got white code in 2.9%, green in 77.6%, and yellow in 12.2% cases (Figure 3B). The median stay at the hospital in patients *with* neurological symptoms was 5 days (mean, 6 days), while in the second group, a median of 5.5 days (mean, 4 days) was observed.

Patients *with* neurological symptoms showed a higher frequency of GI symptoms (*p* = 0.0018). A comparison of the occurrence of pre-existing medical conditions between the two groups resulted in a *p* = 0.62 (>0.05).

### Long-Term Clinical Course

Four (12.5%) patients of the group *with* acute neurological symptoms reported persisting symptoms, which required new admission to our centre. Two cases were readmitted due to dyspnoea and recurrent bronchospasms, respectively. Two 14-year-old adolescents complained about persistent asthenia: the girl had a history of anxiety disorder under pharmacological treatment, while the boy also reported recurrent headaches and arthralgia of the shoulders and elbows.

## Discussion

Rating the prevalence of COVID-19 symptoms in the pediatric population may be subjected to case detection differences, and yet, after more than 2 years since the start of the pandemic, the real impact of SARS-CoV-2 infection on this population stays quite elusive (33). Most of the other published COVID-19 pediatric series have concentrated on the serious multisystem inflammatory syndrome in children (MIS-C) with neurological symptoms or other rare neurological sequelae in children with pre-existing neurological problems.

We analyzed the signs and symptoms of children who tested positive for SARS-CoV-2 with a molecular swab. Notably, in our cohort, the headache was the most frequent manifestation, followed by altered awareness/confusion, and ageusia/anosmia. Even considering the higher age (median, 10.9 years) of patients within the neurological group, ageusia and anosmia were less common in our cohort as compared to the literature (34). Notably, in the group *without* neurological symptoms, the median age at admission was significantly lower, possibly underlying one of the limitations of the current study, as patients aged less than 6 years are expected to have more difficulties in reporting symptoms.

Comparison of the occurrence of pre-existing medical conditions between groups did not result in a statistically significant difference (*p* = 0.62), meaning the occurrence of neurological symptoms could not be affected by previous medical history. Nevertheless, there seems to exist a difference between groups, being respiratory comorbidities the primary pre-existing condition in the subgroup of patients *without* neurological symptoms. Conversely, a high statistical difference (*p* = 0.0018) was found in the concomitant occurrence of GI symptoms within the neurological subgroup.

Only one red priority code was assigned at the ED, pointing toward a low-grade infection severity in our pediatric population. No patient died, and life-threatening events occurred in one patient only (of the “*with* neurological symptoms” group). This data may significantly differ from the current literature on COVID-19 in children, possibly related to the selection criteria of our series, where only patients with a positive molecular swab were included, thus, skimming patients with MIS-C, which usually occurs 4–6 weeks after SARS-CoV-2 infection (35). Moreover, given the Italian health system structure, ED access occurs earlier than in other countries (e.g., the United States), where it is often delayed (36). Accordingly, early hospitalization of patients affected by MIS-C has been related to a better outcome (35). No differences in treatments, laboratory test results, and mean stay at the hospital were found between the two groups of patients. Only two ascertained cases of long-term neurological symptoms were observed at a one-year-long follow-up involving all the patients *with* neurologic features, thus, highlighting the rarity of these complications in the pediatric population (37).

Few reports of real-time PCR (RT-PCR) SARS-CoV-2-positive children experiencing epileptic seizures are currently available in the literature (29, 38–41). In our cohort, 2 patients were presented at the ED with seizures; one experiencing seizures re-exacerbation despite being previously well-controlled with a single ASM, the other with newly-onset seizure within the context of a malacic region due to a previous ischemic stroke. These findings are in line with the literature, indicating that seizures remain a rare presenting symptom in pediatric patients (42, 43), and that a certain degree of predisposed background is necessary to generate epileptic discharges. RT-PCR, for the identification of specific variants of SARS-CoV-2 was not performed as tests preceded the spread of the Delta variant in Italy and the identification of the Omicron variant. Yet, a higher prevalence of seizures has recently been reported in patients affected with these two SARS-CoV-2 variants (44).

In conclusion, neurological symptoms including peripheral facial palsy, encephalitis, and Guillain-Barrè syndrome are rare acute presenting symptoms in children with COVID-19, while they are more frequent at long-term follow-up and within the context of MIS-C (34, 45–48). Some limitations may be found in the current study, including those about a single-centre experience; moreover, the frequent changes in internal protocols (e.g., swab execution indications, clinical management of patients with SARS-CoV-2, the absence of neonates due to different diagnostic pathways) inevitably influenced data collection. Moreover, in line with previous studies, symptoms may have been underreported in younger children. Our study provides a whole-year picture of the acute symptoms in children tested postive for SARS-CoV-2, suggesting that patients *with* neurological symptoms neither have more severe clinical conditions nor have more pre-existing comorbidities. The course of the infection seems quite benign in children; however, additional research including the characterization of the clinical spectrum related to spreading variants (i.e., Delta and Omicron) is needed to fully assess the neurologic implications of SARS-CoV-2 infection in this population.

## Data Availability Statement

The raw data supporting the conclusions of this article will be made available by request to the corresponding author.

## Ethics Statement

The studies involving human participants were reviewed and approved by Comitato Unico Regionale Liguria. Written informed consent to participate in this study was provided by the participants’ legal guardian/next of kin.

## Author Contributions

GP and AR: study design and data acquisition, analysis, and interpretation of data, and wrote the manuscript. FB, MB, NB, EA, MSV, and TG: data collection, contribution to the manuscript, and interpretation of the data. AS, AV, EP, PI, and MM: critical revision of the manuscript. PS and GB: study design and supervision. All authors contributed to the article and approved the submitted version.

## Conflict of Interest

AR has received honoraria from Kolfarma s.r.l, Proveca Pharma Ltd., and PTC Therapeutics. PS has served on a scientific advisory board for the Italian Agency of the Drug (AIFA); received honoraria from GW pharma, Kolfarma s.r.l., Proveca Pharma Ltd., and Eisai Inc.; received research support from the Italian Ministry of Health and Fondazione San Paolo. The remaining authors declare that the research was conducted in the absence of any commercial or financial relationships that could be construed as a potential conflict of interest.

## Publisher’s Note

All claims expressed in this article are solely those of the authors and do not necessarily represent those of their affiliated organizations, or those of the publisher, the editors and the reviewers. Any product that may be evaluated in this article, or claim that may be made by its manufacturer, is not guaranteed or endorsed by the publisher.
