# Supplementary figures and images for: Acute Neurological Presentation in Children With SARS-CoV-2 Infection
Source: Front Pediatr. 2022 Jul 11;10:909849. doi: 10.3389/fped.2022.909849 (PMC9309333; doi:10.3389/fped.2022.909849)

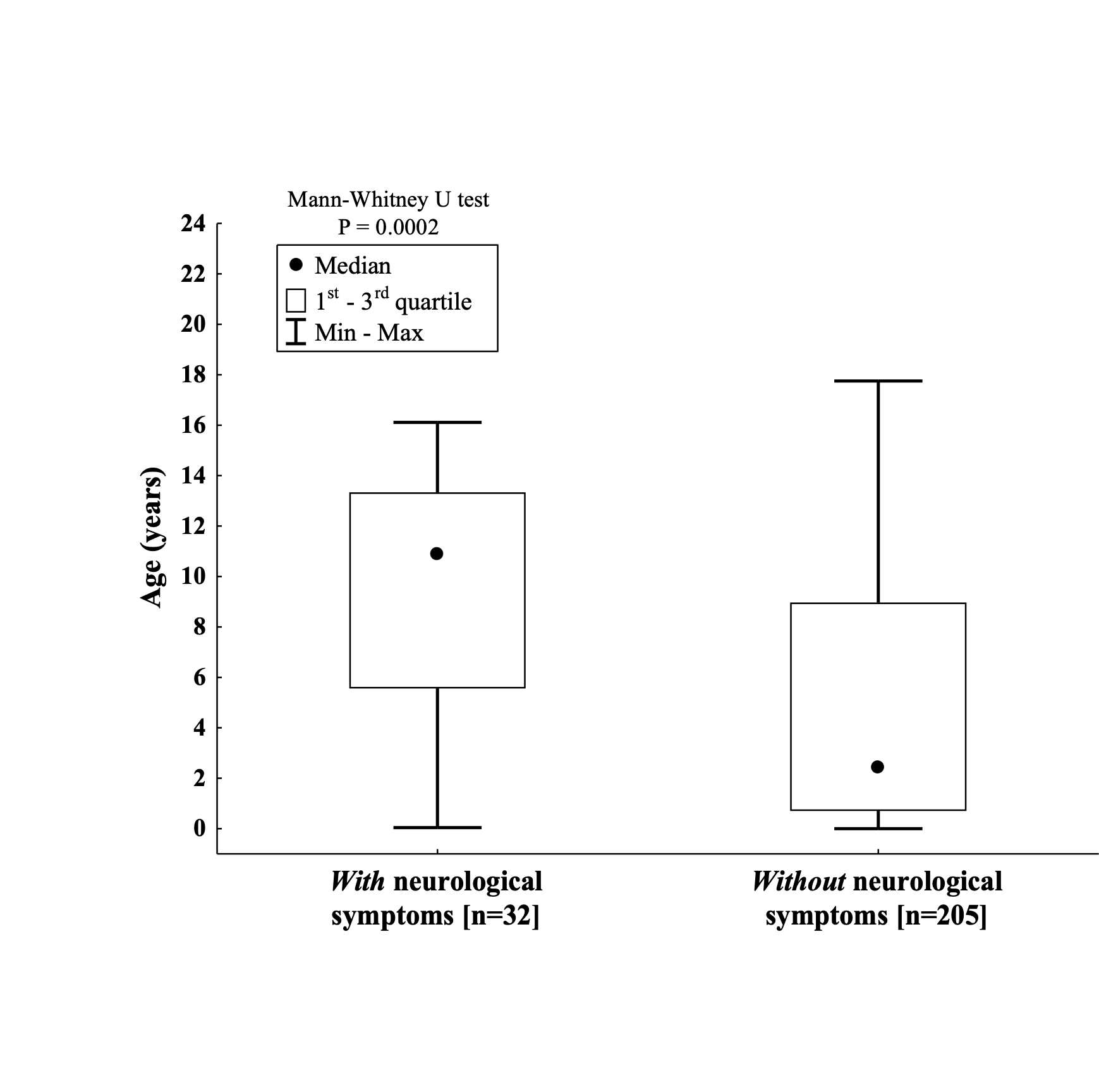

Supplement: Supplementary file 1 [file Image_1.TIFF]
